# Students’ Perceptions of Staple Elements of the Doctoral Program: Research, Courses, Exams, and Seminars

**DOI:** 10.1021/acs.jchemed.5c01358

**Published:** 2026-05-08

**Authors:** Jordan Harshman, Brittany Busby, Erhunmwense Obayuwana

**Affiliations:** † Chemistry Department, University of Iowa, Iowa City, Iowa 52242, United States; ‡ Department of Chemistry, Michigan State University, East Lansing, Michigan 48824, United States

**Keywords:** Graduate Education, Chemistry Education Research

## Abstract

For decades, the core elements of doctoral programs (dissertation research, courses, exams, and seminars) have been used to train independent scientists and prepare them for their careers. Despite this, a lack of systematic job outcome data and a saturated academic market have led to sharp criticism from major organizations regarding the effectiveness of these programs. While previous student surveys have shown general satisfaction, they fail to provide insight into the perceived benefits of each individual program element. This study addresses that gap by characterizing student perceptions of these staple elements, which is crucial for evaluating their efficacy and understanding student engagement. We developed a survey based on a prior qualitative study to measure chemistry doctoral students’ perceptions of the benefits of four key program components. Distributed to over 6400 students, the survey yielded a final sample of 837 participants for quantitative analysis and a subset for qualitative coding of open-ended comments. Quantitative results revealed few differences in student perceptions across their year in the program, division, or career interest. Qualitative analysis showed that while students acknowledged the potential to gain knowledge and skills, their perceptions were largely influenced by the element’s perceived relevance to their careers, the quality of instruction, and, for exams, the mental stress involved. Ultimately, this study found that students expressed general satisfaction with their programs while simultaneously holding cynical views. Their perceptions are narrowly focused on the relevance of each element to their specific research and career goals, reinforcing the critique that doctoral education often prioritizes hyperspecialization (depth) over a broader, more valuable skill set (breadth).

## Graphical abstract

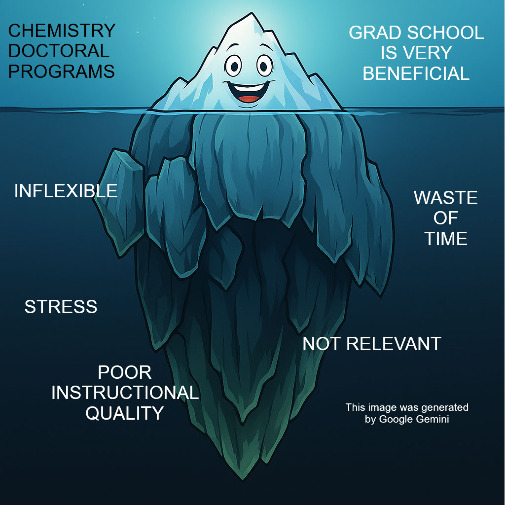

## Introduction

The staple elements of the doctoral program—dissertation research, basic and advanced courses, seminars and colloquia, and candidacy examinations—have existed for decades.^1,2^ These elements are responsible for accomplishing the primary goals of the doctoral program and are universally recognized across most doctoral programs.^3,4^ Together, they have the mission to carry out the primary goals of the doctoral program, which recent studies reveal to be one (or both) of training independent scientists and/or making students competitive for their future careers.^5^

Pertaining to the latter goal, the approximately 3000 chemistry doctorates awarded per year^6^ have saturated the academic job market, which has stagnated around 20 000 total post-secondary faculty positions in chemistry (research and teaching) for the past decade.^7,8^ Domestic and international tracking of job outcomes for chemistry doctoral recipients is not systematically conducted, making any assessment of the true job placement of those with newly minted Ph.D.’s more or less unknown. This uncertainty in job placement has led many organizations, such as the American Chemical Society (ACS),^2,9–14^ National Research Council (NRC),^15–22^ Council of Graduate Schools (CGS),^23–26^ National Science Foundation,^27,28^ and President’s Council of Advisors on Science and Technology^29^ to have sharply criticizing remarks about doctoral programs’ ability to adequately prepare the next generation of advanced chemists.

### Structure and Theoretical Goals of the Staple Elements

We have conducted extensive work to characterize the precise goals of each element found in the modern Ph.D. chemistry program according to faculty.^30^ The following briefs the most observed goals of each element to contextualize what faculty expect students to gain from the staple elements in a doctoral program:

#### Dissertation Research

Research leading to a dissertation is clearly the most quintessential element of the Ph.D. program, and most faculty viewed it as being responsible for engendering a host of knowledge and skills useful in creating independent scientists competitive for future careers. The experience of students varies significantly from advisor to advisor, but structurally, students are expected to develop an expertise in a narrow area of chemistry, carry out, and disseminate a novel investigation. The most commonly listed benefits to students from the multiyear experience are chemistry knowledge, critical thinking, problem solving, and communication skills. Of note, the advisor–advisee relationship could also be considered a staple element of the Ph.D. program, but we include these relationships within the dissertation research experience.

#### Courses

Students take basic and advanced courses with widely varying degrees of flexibility and requirements for the primary purposes of gaining technical knowledge and ensuring that all chemists have a reasonably uniform set of chemistry knowledge. These are expected to benefit students’ in both their degree progression and help them secure future careers.

#### Candidacy Exams

The formal examination system, including both written and oral components, was described by faculty as having two primary purposes. The summative side of the examination system was viewed as allowing faculty to terminate the degree progression of underperforming students or those who did not demonstrate adequate gain of research skills to continue. The formative side of the examination system was viewed as providing students with evaluative and actionable feedback to improve their communication, planning, and critical thinking skills.

#### Seminars

The types of seminars/colloquia were 2-fold: those that required students to present some form of research or literature review and those that required students to attend another experts’ research presentation. The former was primarily seen as a means for students to gain communication skills, while the latter was viewed as increasing technical knowledge, communication skills, and networking skills.

### Students’ Perceptions of the Doctoral Program

A common shortcoming in assessing doctoral education is the noted lack of evidence to argue the efficacy of the doctoral program.^22,31,32^ Throughout a student’s approximate five-year timeline, the only formal data collected to provide evidence of what is and is not working may consist of course artifacts, feedback from a single written/oral exam, brief annual evaluations, and a dissertation. With limited information to evaluate the benefits of the current doctoral program, a valuable source of information regarding a doctoral program is the perceptions from the students themselves as they transition into their chemistry careers.

Prior ACS surveys of chemistry graduate students suggest high (78%–79%) general satisfaction with their overall programmatic experience.^33,34^ Similarly, a 2015 ACS survey^35,36^ of 784 Ph.D. chemists agree, on average, that they were satisfied with their overall graduate education. This majority-positive perception from students appear to conflict with the sharp criticism from national organizations and hundreds of chemists over the past several decades.^32^ However, the same reports do not provide the perceived benefits from each individual element of the graduate program, as this was not the intention of the past attempts.

We believe that there is great value in characterizing student perceptions of the staple elements of doctoral programs, as it allows an evaluation of these elements when efficacy data are sparsely available. Furthermore, it is generally accepted from fundamental learning theories^37^ that to get the greatest, or any, educational benefits requires student buy-in to the instructional strategies. We predict a wide range of individual perceptions will be observed and hypothesize that student perceptions of these staple elements *may* differ based on experience in the program, specific chemistry area due to divisional norms and expectations, and career interest. Related to experience in the program, we previously showed how some graduate students transition from open and inviting perceptions of program elements to deep skepticism and cynicism after a few years of experience in the program.^38^ In this study, we also found that graduate students tend to value knowledge if it is directly related to their dissertation projects and justify the benefits or perceived time wasted in terms of what will help them succeed in the program and in their careers. While this study provides an exploratory beginning, it was a case study that cannot be generalized across the graduate student population. Additionally, because each traditional chemistry division represents mild to moderately different knowledge bases and norms from others, it is plausible that certain elements of the Ph.D. program may be more valuable than others. Similarly, past research has shown that graduate students’ perceived value of educational activities can nearly exclusively depend on the perceived relevance to their career goals. However, due to a lack of previous investigations, we can only hypothesize that student perceptions *may* meaningfully differ based on experience in program, division, and/or career interest, but do not have sufficient reason to predict *how* they might differ, if at all.

### Research Questions

This study investigates the following research questions:

(1) To what extent do graduate students perceive gaining benefits from the four staple elements (research, courses, candidacy exams, and seminars) of the doctoral program?
  (a) How do these perceptions meaningfully differ across students’ experience in the program, closest related chemistry division, and career interest?
(2) What informs students’ reasoning behind their perceptions of the staple elements?

## Methods

### Survey Development

A survey was developed based on a previous longitudinal study examining growth in graduate students.^38^ While the survey had multiple purposes, for the portion described here: *This survey was designed to measure US chemistry graduate students’ perceptions of benefits gained from staple programmatic elements in doctoral programs*. In the first section, we verified that participants were graduate students enrolled in a doctoral chemistry program and collected their current career interests, year in the program, primary division of study, elements of their current doctoral program, courses taken, frequency of seminars attended, and papers published or submitted. Next, graduate students were asked to select the appropriate agreement/disagreement on a 6-point Likert scale (with a “not applicable” option) to the statement: *Knowledge and/or skills I have gained from this component will be beneficial to me*. The six options consisted of “Strongly Disagree,” “Disagree”, and “Somewhat Disagree” and then the same for agreement options.

In the statement, “this component” referring to each of the staple elements of the doctoral program, but these were presented as an expanded version of the four elements described: courses, dissertation defense, candidacy exams (written portion), candidacy exams (oral portion), seminar, the requirement to publish a paper, the requirement to present at local or national conferences, and research. This expanded list was used to capture greater breadth, but later questions relied only on the four staple elements, as the survey was already getting too long and risked participant burnout. The following questions were only split across the four staple elements and students were asked the following stems on the same 6-point agreement Likert scale:

- This component has been/will be a waste of my time. I would rather use the time toward something else.
- I gained/will gain transferrable skills from this component.
- I gained/will gain knowledge from this component that will help me succeed in obtaining my desired career.

These questions were chosen because in the previous study’s interviews, students framed the majority of their perceptions by what benefits, or lack thereof, came from each element.^38^ Additionally, we noted that all students in the case study held complex, often contradictory perceptions and included many critical aspects while simultaneously recognizing the benefits of doctoral programming. This is why we chose positively and negatively framed as well as career-framed items. After responding to each question, participants were then reminded of both responses and asked to justify (open-ended textbox) why they responded the way they did.

These comments were collected separately for each staple element and qualitatively analyzed with an open-coding approach. Ideas expressed in the code were generalized and written down with a brief description, which was updated when similar ideas were found. This led to an initial codebook after the first pass of the data, which was refined in the second pass of the qualitative comments. A total of 14 codes expressed positive perceptions, 13 expressed negative perceptions, and two additional codes captured varying or unclear sentiments. For example, one participant justified their perceptions as follows:

“There are few courses that are relevant to my research and the professors teaching those courses went on sabbatical those semesters. I was not able to take the courses that were relevant to my research, and thus I found the course component a waste of time. I enjoyed learning about other topics, but I have not found a use for that knowledge yet.”

Three codes were applied to this quote:

- Negative: Not Relevant to Field (“few courses that are relevant to my research...” and “...haven’t found a use for that knowledge yet.”)
- Negative: No Flexibility (“I was not able to take the courses...”)
- Positive: Gained Skills [Ambiguous] (“I enjoyed learning about other topics...”)

The full codebook with precise code definitions used can be found in the Appendix A in the Supporting Information. No unexpected or unusually high safety hazards were encountered, and the study received institutional approval from the Auburn University IRB STUY00000330.

### Survey Validation

We present evidence of the validity and reliability of interpretations made by examining the data via the following evidence recommended by the Standards for Educational and Psychological Testing.^39^

#### Evidence Based on Response Process

The survey used direct quotes from the prior case study, a form of response process validity evidence as we used the words used by real graduate students to frame the stem (particularly emphasizing “knowledge”, “skills”, “component”, and “beneficial”) and items (e.g., “waste of time,” “gaining knowledge/skills,” “transferrable skills”). Additionally, we included an open-ended question, allowing participants to give feedback about any confusion with questions in the survey. After the first 100 responses, we collected the feedback and made minor adjustments to the text as necessary. Additionally, qualitative open comments provided verification that students understood the prompts and adequately understood the intentions of the questions.

#### Evidence Based on Internal Structure

Prior to answering our research questions about perceptions, we first provided evidence to support our hypothesis that each element is uniquely and separately viewed by graduate students, leading to differing perceptions within each element. Examining confirmatory factor analysis (CFA) models for a single, omnibus model (e.g., perceptions of all elements share the same latent construct) versus four, separate 1-factor models for each element (e.g., perceptions are unique latent constructs for each element) served as the evidence to our hypothesis. Therefore, four separate 1-factor and an omnibus model listing all individual models under one latent construct (perception of doctoral program) were tested (Table 1). Results evidence the validity of interpretations made for the single-factor models as opposed to attempting to measure a single, stable “perceptions of doctoral program” construct. Graduate students have perceptions of each element separately.

**Table 1:** CFA Results for the Five Models Tested

Model	Formula (Item # as presented in the SI)	χ^2^	*p*	CFI	TLI	RMSEA	Omega
Research	Research = 15h + 16a−c	5.49	0.06	1.00	0.99	0.05	0.73
Courses	Courses = 15a + 16d−f	26.70	0.00	0.95	0.86	0.12	0.66
Exams	Exams = 15c + 15d + 16j−l	18.77	0.00	0.99	0.98	0.07	0.66
Seminars	Seminars = 15e + 16g−i	4.13	0.13	0.99	0.97	0.04	0.57
Omnibus	Research + Courses + Exams + Seminars	591.41	0.00	0.89	0.86	0.08	Average: 0.70

We examined the evidence for measurement invariance across the three groupings of interest: experience in the program (pre- vs post-candidacy exam), division (six in total), and career interest (primary interest in academia, industry, government, and other). Related to research, none of the group sets supported configural, metric, or scalar invariance due to convergence issues, lack of variance, and variables with only one level. In contrast, courses demonstrated full measurement invariance (configural, metric, and scalar) across all three group sets, supporting the equivalence of the construct. For candidacy exams, configural invariance failed across all group sets, despite some later steps passing. Finally, for seminars, full invariance was supported only by candidacy status, while field and preferred career groups failed to establish configural invariance. Comparisons across all three groups for all four key elements are included for comprehensiveness (via Kruskal–Wallis tests), but we discourage the interpretation of these tests of differences where measurement invariance is lacking. All statistical analyses were performed in R v4.5.1. CFA was computed using the lavaan package (v 0.6–21), which performs listwise deletion for missing data.

### Sample Description

Of the 6489 chemistry graduate students who were invited to take the survey, 1274 students started it. Responses from 437 students were removed who were either not enrolled in a chemistry doctoral program, did not consent, were not beyond their first year of graduate school, or left more than 19 missing responses. This left a final sample of 837 graduate students. Participants were almost uniformly distributed across years in their program (no first-year students were invited, as they do not have significant experience with various elements). Participating students studied a variety of topics with 13.5%–20.7% listing one of the five traditional divisions of chemistry (Table 2). Personal demographics (Table 2) were also collected to demonstrate the representative nature of the sample, as opposed to any theory that different personal demographics would lead to predictably different perceptions. We received open-ended justifications from 252 participants regarding seminars, 213 regarding courses, 204 regarding exams, and 169 regarding research.

**Table 2:** Sample Demographics

Years in Ph.D. Program	Count	Percent
2nd Year	167	20.0
3rd Year	184	22.0
4th Year	171	20.4
5th Year	169	20.2
>6th Year	146	17.4
		
**Field**		
Analytical	113	13.5
Biochemistry	153	18.9
Education	35	4.2
Inorganic	127	15.2
Organic	173	20.7
Physical/Computational	158	18.9
Other	78	8.6
		
**Gender Identity**		
Female	425	50.8
Male	362	43.2
Nonbinary	13	1.6
Did not disclose	36	4.3
		
**Preferred Career**		
Industry	301	36.0
Academia	215	25.7
Multiple, Do not Know, or Other	200	23.9
Government	95	11.4
Did not respond	26	3.1
		
**Age**		
20–29 years old	709	84.7
30–39 years old	101	12.1
40–49 years old	10	1.2
50+ years old	12	1.4
Prefer not to answer	5	0.6
		
**Ethnicity**		
Caucasian	511	61.1
Asian	207	24.7
Latino or Hispanic	64	7.6
Prefer not to answer	38	4.5
African-American	36	4.3
Other/Unknown	26	3.1
Native American	7	0.8
Native Hawaiian or Pacific Islander	5	0.6

The careers that students were interested in are shown in Figure 1. Students in the sample were moderately or very interested in careers in industry (86%), government (84%), teaching-emphasis professor (60%), and research-emphasis professor (54%), which are consistent with other estimates of career desires among doctoral students.^33,34^

**Figure 1:**
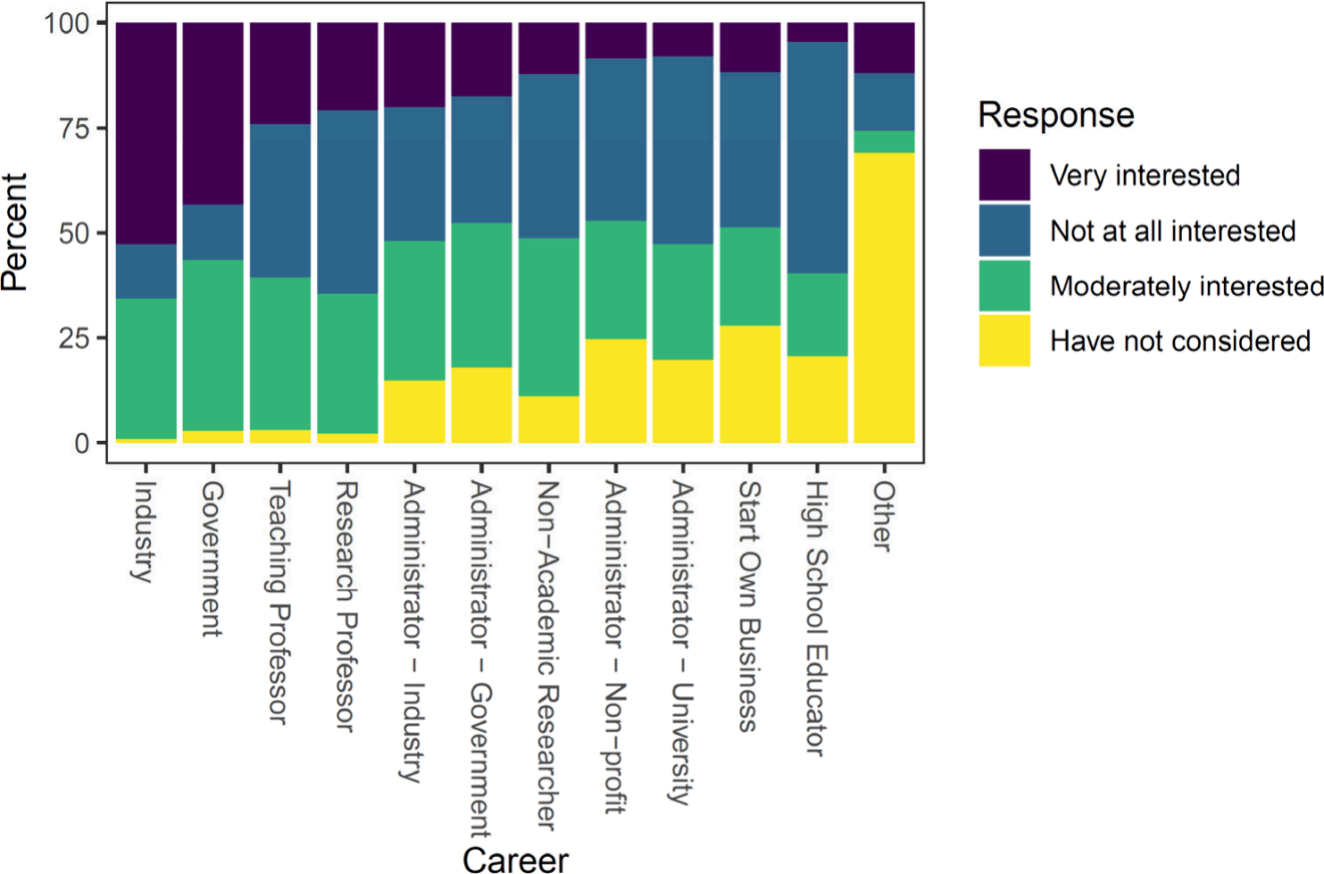
Career interests of the sample of doctoral students.

## Results and Discussion

Results and interpretations are organized by research question where the Likert responses answer Research Question 1 (perception of benefits from staple elements) and Question 1a (differences in those perceptions across groups). Research Question 2 (why elements are beneficial or not) is answered by open-ended comments.

### Research Question 1: Perceptions of Benefits from Staple Elements

The most direct answer to this question is given by the distribution of agreement to key statements for each element, provided in Figure 2. The majority of chemistry graduate students agreed to some capacity that all elements were beneficial, with seminars receiving the lowest agreement (85.3%) and research receiving the highest (98.7%, Figure 2A, summation of responses to the right of 0). Graduate students also generally agreed that they gain knowledge from elements that help achieve their desired careers (Figure 2C) and gain transferable skills from programmatic elements (Figure 2D). Research was perceived the most positively across all statements with more than 90% of students expressing somewhat to strongly positive agreements (research is beneficial, helps succeed in career, and provides transferrable skills) or disagreement (is a waste of time). As found in other studies,^33,34,40^ these positively skewed response distributions support the notion that graduate students hold positive perceptions about their experiences in doctoral education.

**Figure 2:**
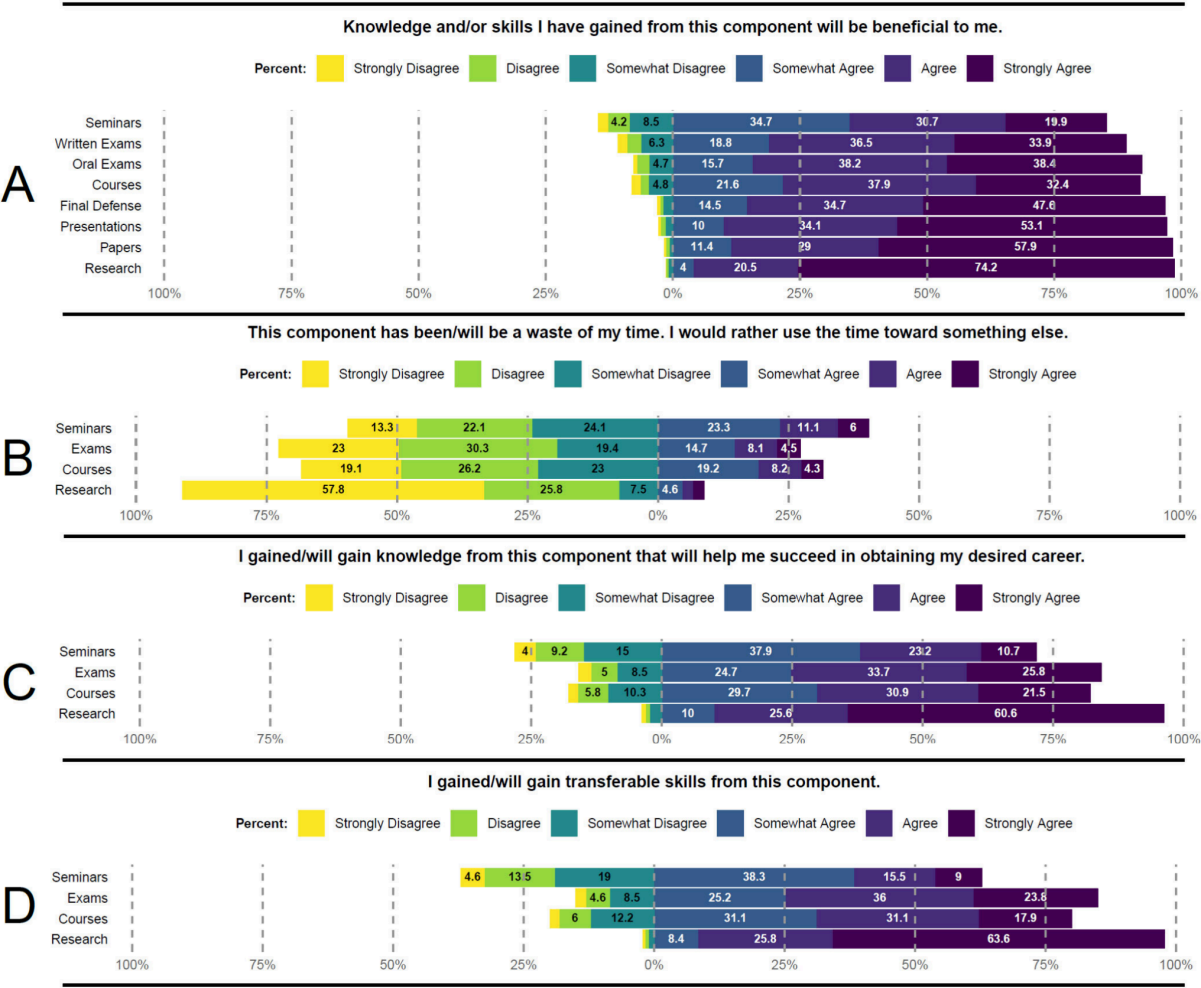
Percent of students responding to four statements (exact wording provided above each graph). In panels (A), (C), and (D), strongly to somewhat agree (blue) indicates positive perceptions; negative perceptions in (B).

However, a significant number of students also agreed that various elements were a waste of time or their time would be better spent elsewhere. Of the sample, 40.4% of the students saw seminars as a waste of time (Figure 2B, summation of responses to the right of 0), 31.9% agreed the same about courses, and 27.3% about exams. Seminars, courses, and candidacy exams also drew skepticism from students, as they were more likely to somewhat to strongly disagree that they provided transferable or career-related skills. These results imply that many, but not a majority, of graduate students have significantly negative perceptions about various elements of the doctoral program, with the exception of research.

The reasons why students perceive elements as generally beneficial but simultaneously see many as a waste of time are explored further in Research Question 2a. This implies that graduate students have mixed perceptions about the utility of various components of the graduate program, a sentiment echoed in many discussions about doctoral programs’ capacity to prepare the next generation of scientists.^14,15,22^

### Research Question 1a: Differences in Perceptions Across Groups

To test if the response distributions in Figure 2A (“*Knowledge and/or skills I have gained from this component will beneficial to me*”) differ when broken down by certain groups of students, we present the same plots for every individual group. Additionally, we broke down elements further by splitting the written and oral components of exams and adding a requirement to publish papers, present research, and defend a dissertation as an element. Figure 3 shows the distribution of responses broken down by experience in the program (pre- versus post-comprehensive examination). Similar breakdowns by primary division (Appendix Figure 1) and primary career interest (Appendix Figure 2) are placed in the Supporting Information for space concerns. While we conducted inferential tests for differences across groups (Appendix Table 1), we base our conclusions primarily on the descriptive figures on account of limited measurement invariance evidence. If the distributions are reasonably similar across groups, there is no evidence that characteristic determined the likelihood of finding a specific element beneficial or not.

**Figure 3:**
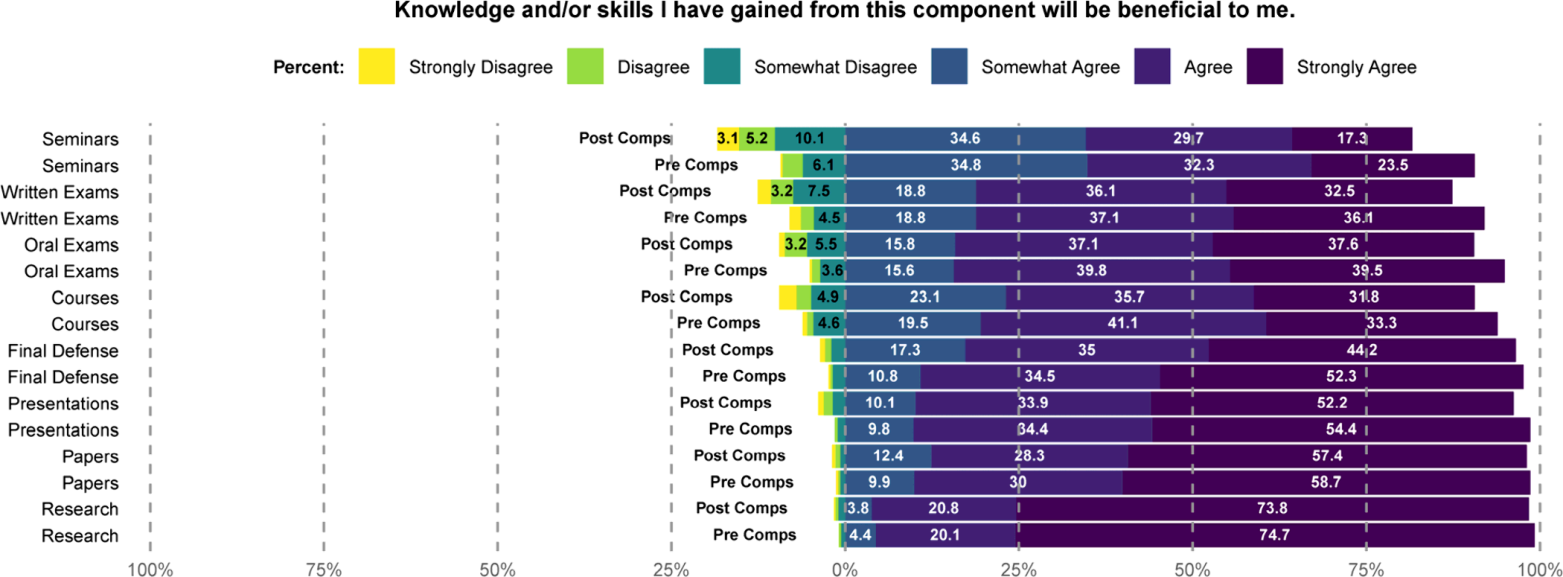
Response distribution of agreement to perceived benefits of each component, broken down by pre-/post-comprehensive exam (comps) status.

For example, comparing the distribution of responses for the Pre Comps students to the Post Comps students across written and oral exams, final defense, presentation and publication requirements, and research (Figure 3) shows a nearly identical distribution in each response option, confirmed by nonsignificant tests of ordinal differences (Appendix Table 1). In contrast, more Post Comp students appear to disagree that seminars are beneficial than Pre Comps students (top two distributions in Figure 3), and this is confirmed by a significant test of ordinal differences (Appendix Table 1, row “15.e”). For this reason, we conclude that, with the exception of seminars, graduate students’ time in the program does not lead to meaningfully different perceptions of staple elements.

Examining Figure 3, Appendix Figures 1 and 2, and Appendix Table 1, across all three students’ characteristics examined (pre- and post comprehensive exam, primary division, and career interest), no significant differences were observed for any element except seminars. Students who have passed comprehensive exams or study education research reported higher levels of disagreement that seminars were beneficial. In contrast, only organic and inorganic students expressed more positive perspectives about seminars. However, given the uncertainty in the measurement invariance that supports testing across groups, the overwhelming similarity of distributions across groups, and the lack of a theory that can explain why different groups would hold systematically different perceptions, we conclude that perceptions do not likely differ across student experience, field, or career desires.

### Research Question 2: Why Elements Are/Are Not Perceived To Be Beneficial

Figure 4 shows the frequency of codes that were observed in the submitted open-ended comments. Each code is colored by its sentiment (positive, negative, or neutral). Each element received a different number of comments, so Figure 4 only shows the codes that were observed in greater than 3% of the comments received for that element. For each element, a subsection is dedicated to discussing the frequencies of observed codes (Figure 4) followed by insights gleaned from a qualitative analysis of submitted comments.

**Figure 4:**
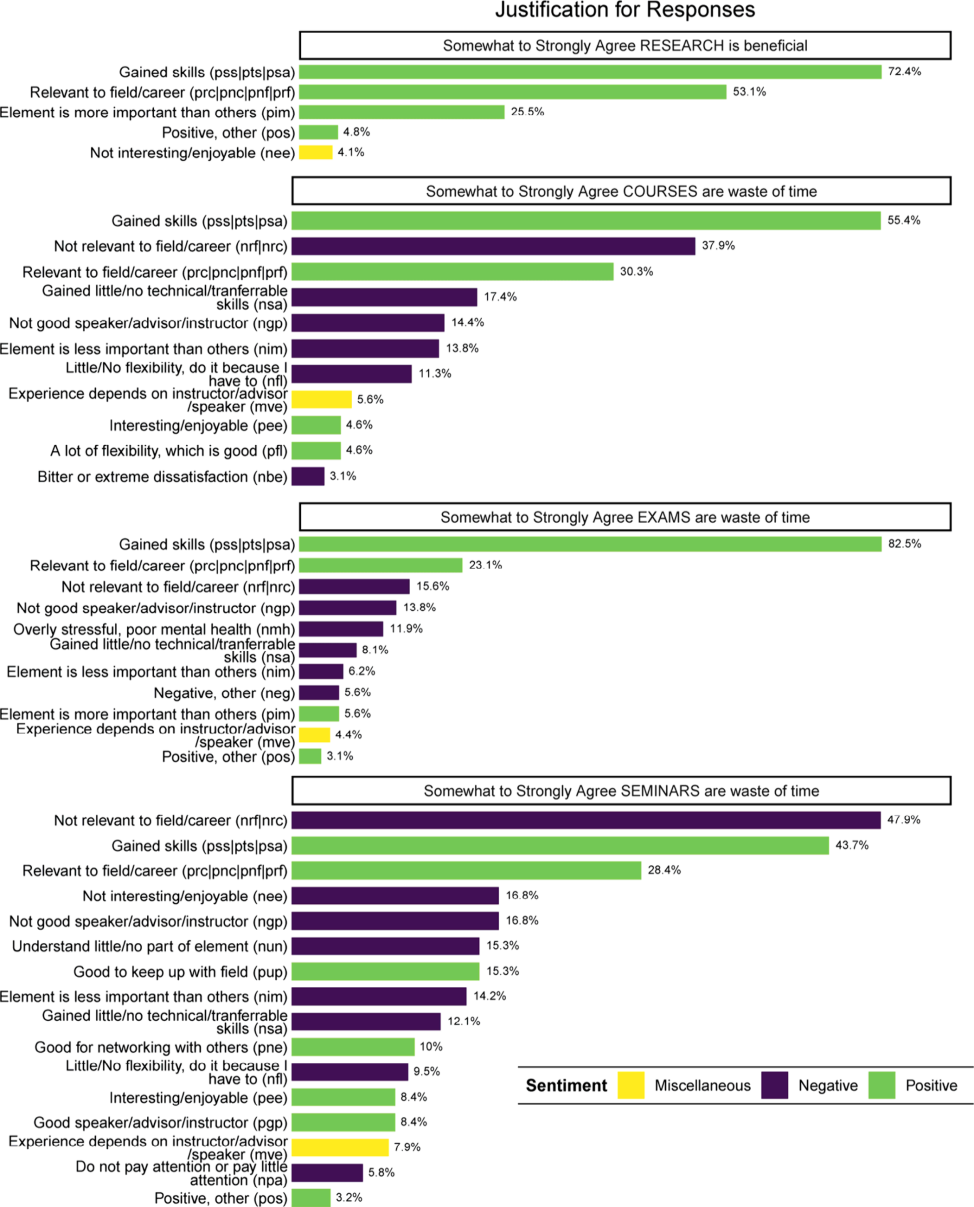
Most commonly observed rationalizations for graduate students’ perceptions for each staple element.

#### Research (169 comments)

Because research was viewed positively, it is not surprising that most comments submitted were coded positively. Of the comments submitted, 72.4% referenced a gain in knowledge or skills, 53.1% noted that research was more relevant to their field or career than other elements, and 25.5% saw research as being more important than other elements in the program. Example comments like “*...you will learn how to imagine, start, evaluate, and complete this target. This is invaluable to all future work and research*” demonstrate students’ positive view of the skills gained in research and the importance it carries. Additionally, phrases such as “*will be useful in pursuing various careers*” demonstrated the perceived utility of those gains.

#### Courses (213 comments)

55.4% of comments received about courses mentioned gaining skills and 30.3% claimed courses were relevant to their field or career, in contrast to 37.9% of comments that claimed the opposite. This coincided with the 11.3% of comments made about the lack of flexibility students had in course selection: “*Not all courses are good. Some provide excellent relevant information and skills for future careers. Some are not relevant at all but certain schools mandate having 6*”. Negative responses such as these highlighted palpable frustration largely centered on inflexibility in course selection, relevancy to students’ studies/career goals, and inadequate instructional quality. *Recommendation:* We recommend loosening course requirements and/or providing greater flexibility in the available courses to meet certain requirements. The majority of knowledge and skills gained throughout a doctoral program will not occur in the courses students take but rather through their research experiences and interactions with their group. Additionally, we should not ignore the incentives that faculty have in designing and conducting graduate courses that *can be* (but are not always) more convenient, smaller, and less time-consuming to teach, compared to traditional undergraduate courses. If these benefits are present, they should not be the primary reason a graduate course is offered when alternatives like smaller workshops and seminars could be used to teach nuanced knowledge and skills, allowing students to explore a greater variety of courses.

Many students also expressed awareness and concern of instructor quality, with 14.4% directly claiming the instructors were of poor quality and 5.6% implying the same by saying that their experience depended on the instructor:

“Some classes were just filler to meet requirement. Other classes were taught by very old professors, so the techniques/knowledge taught was outdated/no longer practice. Some classes was taught base opinion of the professor (what they believed to be important even if it’s irrelevant).”

Comments like “the quality of instruction at [university] is poor” and “they really are not designed for learning, but just cranking out tasks to get a grade” highlight how much students pay attention to how professors approach designing and implementing their graduate courses. *Recommendation*: Faculty should undergo basic pedagogy training if they have not already done so. The advanced content nature of graduate courses likely makes it look different than many undergraduate courses, but a basic understanding and application of learning theories, pedagogical strategies, classroom management, and teaching technology proficiency should be standard practice in graduate courses.

Courses were also the staple element to observe the largest frequency (3.1%) of comments characterized as bitter and overly negative. This naturally subjective determination arises when participants spoke in strong opposition to the element, such as “but then they also want me to take another f***ing survey class when past experience indicates strongly that they’re a waste of time so I’m a little peeved” and:

“It was obvious that the professors did not care about the course. They were unprepared for class a lot of times because of this, and they were not very willing to help when students who were struggling... It seemed as though the courses were a formality and a way of filtering out students from the program.”

#### Exams (204 comments)

Perceptions of candidacy exams were rationalized very similarly to how courses were, with frequent allusions to gaining skills from exams (82.5%), a mix of being relevant (23.1%) versus not relevant (15.6%) to their fields/careers, and observing a lack of (13.8%) or wide variety of (4.4%) instructional quality. With exams, instructional quality refers to how professors wrote the exams and their expectations with perceived failure to adequately portray expectations (e.g. “*this process felt like I couldn’t hit water if I fell out of a boat*”) and:

“[the expectations placed by the professor] made the written exam a complete waste of time, effort, and experience. As it made the exam needlessly vague, hard, and frustrating.”

This highlights the importance, from a student’s perspective, of being adequately prepared and aware of the expectations for candidacy exams. *Recommendation:* Candidacy exams should be designed with a clear purpose. If the exam is supposed to be summative (e.g., primarily/exclusively for the evaluation of performance), professors have an obligation to be very clear about expectations so that students can adequately prepare. Related, all committee members should be consistent in evaluating students based on those expectations. If the exams are meant to be formative (e.g., primarily/exclusively so that students may learn from the experiences), then the stakes of the exam and stressful nature of the experience should be attenuated. Frequent feedback prior to the candidacy exam would go a long way in assuring students that they are safe, yet still maintain the intellectual rigor of the examination process. Finally, it is common practice that students must complete the exams completely independently. We question the logic of this requirement as students have likely never written a research proposal (novel or their main dissertation project) at the stage of the exam. Expecting them to perform adequately on their first try without guidance, sometimes without ever having seen a single research project through to completion, is likely well outside the zone of proximal development and may lead to poor educational outcomes.^47^

A unique code that came up significantly related to exams and no other staple element was the notion of an unnecessarily stressful, harmful to mental health experience (11.9%):

“I think that candidacy exams have become a meaningless academic hazing ritual...the candidacy exams are (ab)­used by PIs as a way to ferret out the people they no longer want to support. I didn’t learn anything from mine that couldn’t have been learned another (less traumatizing) way.”

“They say this exam is supposed to be to help you think under pressure, but the scenario is not realistic at all. I will never be in a room again where people are TRYING to mess me up so aggressively.”

Many of these codes discussed the learning gains that were plausible, but interrupted by the stressful nature of the experience, while others treated it as a neutral component that did not alter the positive aspects:

“Candidacy exam was brutal and boarderline [sic] traumatic in it’s requirement for wholistic knowledge of an entire field. That standard, however, really forced me to evaluate my own project in the field and greatly increased it’s scientific rigor.”

*Recommendation*: Faculty should be keenly aware that the high stakes students face when entering a candidacy exam have a significant potential to interfere with learning and performance. If students are focused mostly on the fear of being kicked out of the program, losing their immigration status, having to relocate, and other life-changing consequences, it is not surprising that they may not be paying close attention to the learning opportunities possible in the feedback provided. Following the previous recommendation by providing clear expectations in advance will likely go a long way toward attenuating stress felt by students and improving learning outcomes.

#### Seminars (252 Comments)

In contrast to courses and exams, the most frequently observed code in submitted comments was negative. 47.9% of comments claimed that seminars were not relevant to students’ field/careers while only 28.4% claimed the opposite. Additionally, only 43.7% mentioned gaining direct knowledge and skills, substantially lower than all other elements. 16.8% of student comments reference poor presentation skills from speakers while an additional 7.9% said the experience varies speaker to speaker: “*some speakers are not very good at targeting a talk to the level of the audience*” and “*The speakers invited to give presentations are not well screened for the caliber of their presentation skills*.” Of note, many who perceived limited presentation skills still found that useful: “*You can learn a lot from watching both good and bad presentations*.” Similar to “bad” presentation styles, 16.8% of comments also rationalized their perceptions in terms of the limited enjoyment or interest they got from the seminar: “*Many talks cover topics which aren’t interesting or thought provoking*.” This likely led to the 5.8% of comments that explicitly mentioned not paying attention at all during seminars:

“If anything, I think colloquiums and seminars have taught me to be quiet and let me daydream for at least an hour. Most of the speakers I have seen this year are poor presenters/communicators and do not present material that I am able to understand.”

Unique codes related to seminars included the 15.3% of students who reported seminars allowed them to keep up with the field (e.g., “*seminar still has some use to give...an indication on what academic peers are working on*”) and 10.0% who claim that seminars provided good networking opportunities for students: “*The seminars can however provide a platform to network if the presenter happens to be within your field of research*...”

*Recommendation*: Students took issue with the relevancy of invited speakers and the lack of presentation skills by many of the chemists but found it valuable to get a sense of what is being done in other fields and to network with established researchers. Given the sharply critical views found, if students are required to attend seminars, departments should carefully weigh the realistic benefits that students will gain from attending with the costs, mainly students’ time. Speakers are likely invited based on many motivations that do not have the students’ best interests. This is not a bad thing, as research institutions will thrive by welcoming new ideas and experts with skills and resources unavailable at their home institution. However, the niche nature of many traditional speakers will naturally limit the amount of knowledge and skills gained. A way to increase the impact of speakers would be to encourage or require all invited speakers to incorporate some elements into their talks (likely reducing “how much” of their results can be shared), such as

- Taking significant time to summarize the broader impacts of their research problem
- Explaining the background of techniques that may not be familiar to all chemists
- Dedicating a portion of their talk as a “teaching tidbit”, where they formally teach a new technique that would be valuable to the home department
- Limiting the quantity of findings or projects to avoid cognitive overload

#### All Components: Depth versus Breadth in Doctoral Education

Students found mixed and seemingly contradictory perceptions about the benefits of the components (Research Question 1). The ubiquitous presence of “relevance to field/career” codes in the comments provided by students demonstrates that the degree to which students found that element relevant to either their specific dissertation research or career desires determines perceived benefit. We argue there is a good reason for this: the leading theory^41–43^ that explains how students grow is socialization theory,^44,45^ in which one step involves students becoming aware of cultural norms and expectations. Our previous study^38^ documented how rapidly graduate students become aware of and adopt this fixation on “only knowledge and skills directly related to my research area are valued,” thereby demonstrating a value in depth. This notion is also consistent with one of the chief mechanisms for why doctoral education is so commonly criticized as inadequate—a hyper-specialization on depth in a specific area, as opposed to breadth.^14,32^

However, from the student perception, depth is likely valued *because it will be required to progress/succeed in the degree*. To the same extent, students also greatly desire breadth for the purpose of gaining more experiences that will help them be competitive for careers:

“I believe that while the course will be valuable for my current research projects, the knowledge gained from a majority of the course taken will not be as useful for my desired career...”

This demonstrates that students are able to simultaneously value depth for the purpose of succeeding in the doctoral program but separately acknowledge that depth alone will not help them for their careers. This nuanced understanding explains why this student agreed that courses were beneficial (value depth to complete degree) but also somewhat agreed that courses were a waste of time (valued more breadth to be competitive for a career). The role that we as faculty and a culture of chemists play in all four of the staple elements cannot be ignored; it is easy to see where students “pick up” the value on extremely specific technical knowledge and skills. (1) The dissertation research project is designed to present students with an opportunity to be the foremost expert in an incredibly niche area with novelty as a requirement. The novelty requirement of research explicitly values depth versus breadth. (2) Graduate courses, according to many of the comments made by participants and consistent with common experiences, often lack any standards, and academic freedom allows faculty and departments to require/offer courses that cover only a narrow slice of a subfield of a particular division. (3) As evidenced by comments provided, written and oral exams come with the expectation that students must procure accurate and well-articulated answers to extremely specific content questions and are often required to think of a completely novel (and therefore highly specific) project. (4) Most seminars invite/require students from all divisions to attend a speaker where the norm is to give a technical talk *without* dedicating significant time to ensure that the audience is familiar with the specific physical, computational, analytical, and other techniques.

In all of these examples, students can easily get the idea that to be a successful scientist is to focus primarily, or more concerning, exclusively, on a very specific area of chemistry and treat all other content as irrelevant and/or unvaluable. We are not arguing that advanced science will not require very specialized knowledge, only that devaluing knowledge outside of a narrow focus may be to the detriment of the student, considering the goals of doctoral education are to train independent scientists and prepare them for their careers.^5,46^ We therefore encourage designers of doctoral programs (department heads, graduate program officers, etc.) to ensure that elements offer exposure and role models that value the breadth of knowledge and skills.

#### All Components: Adequate Assessment

One key finding this study reveals is that we ourselves and prior studies^33,34^ can find many positive perceptions of doctoral students about their programs at a broad level, but this is not the whole story. To their credit, these prior studies also reported plenty of concerning data coming from graduate students, but we target this discussion on more local efforts in assessing doctoral programs. One primary challenge of doctoral programs is that they tend to lack quality means of assessing how well programmatic elements are working.^31^ If formal evaluation efforts are too superficial, faculty may be given a misleading impression that their major programmatic elements are working as intended. In situations where departments do not have access to assessment data, faculty are even more susceptible to believing the program is without significant limitations as prior research shows that students will likely be hesitant or unwilling to discuss uncomfortable issues with their advisors.^48^ This study shows that, if asked, graduate students will provide very specific reasoning for why they find certain programmatic elements more or less beneficial. Chemistry departments would likely benefit their students by taking these perceptions into consideration when discussing the design of their doctoral programs.

## LIMITATIONS

One of the greatest limitations in this study is that we solicited responses only from currently enrolled graduate students, thus excluding those who had left the program already. This can lead to a survivorship bias and paint a more positively framed perception of doctoral education as those who leave the program, either voluntarily or not, will have experiences different from those choosing to continue in the program. Compared to the national distribution of graduate students, the sample responding to the survey is overrepresentative of the domestic student population. While we have no reason to believe that perceptions of the staple programmatic elements will differ by international status, it does mean that if there were to be systematically predictable differences, our results could mask perceptions held by more graduate students. Finally, our treatment of seminars was to focus only on the attendance of seminars, not on students giving a formal seminar. We did this because the requirements, contexts, frequency, and expectations varied too much from participant to participant, but the attendance requirement and selection of departmental seminars/colloquia was much more homogeneous. This means that we cannot conclude on perceptions of students in the programs that require some form of presenting a seminar.

## Conclusions

Graduate students currently enrolled in U.S. chemistry doctoral programs frequently report gaining knowledge and skills from the four staple elements of these programs: Research, courses, candidacy exams, and seminars. However, these positive perceptions coexist alongside mild to sharp criticisms and limitations of the same elements. Meaningful proportions of graduate students discussed how poor instructional quality, lack of relevancy to their research fields and desired careers, lack of flexibility, and other aspects of staple elements led students to believe certain elements were more of a waste of time and/or less beneficial than they appear when examining general perceptions.

## Supplementary Material



## Notes

Appendix A: Codebook; Appendix B: Tests of Differences in Groups; Appendix C: Measurement Invariance (PDF)
