# Supplementary material for: Students’ Perceptions of Staple Elements of the Doctoral Program: Research, Courses, Exams, and Seminars
Source: J Chem Educ. 2026 May 8;103(6):2843–53. doi: 10.1021/acs.jchemed.5c01358 (PMC13261870; doi:10.1021/acs.jchemed.5c01358)
Supplement: Supplementary file 1 [file ed5c01358_si_001.pdf]

Appendix A: Codebook

Positive Codes

**Code (abbreviation)**	**Description**
Gain technical skills (pts)	Element provides modest gain in **technical** skills, knowledge, training, or experiences. This includes any chemistry, instrumentation, methods, research, and so on but does not include transferrable skills. "Modest" is indicated with no adjective (i.e. "I gained skills") or adjectives like "a lot" or "many", but not "a little" or "few". Should only code one positive/negative skills code in one comment.
Gain transferrable skills (pss)	Element provides modest gain in **transferrable** skills, knowledge, training, or experiences. This includes communication, teamwork, management, life, and other transferrable skills, but does not include technical skills. "Modest" is indicated with no adjective (i.e. "I gained skills") or adjectives like "a lot" or "many", but not "a little" or "few". Should only code one positive/negative skills code in one comment. (pss = positive soft skills to avoid same abb. as pts). Common transferrable skills are teamwork, communication, problem solving, critical thinking, and management. For seminars, if someone says they learn from good and bad presenters, code here as transferrable skills, pgp, and ngp. For exams, this code took on positive affects like "how to work through stressful situations"
Gain skills (ambiguous, psa)	Element provides modest gain in skills, knowledge, training, experiences, but it is unclear whether they are transferrable or technical (i.e. "I gained skills"). "Modest" is indicated with no adjective (i.e. "I gained skills") or adjectives like "a lot" or "many", but not "a little" or "few". Should only code one positive/negative skills code in one comment. Need to read the whole comment to determine if a broad statement (e.g. "I learned skills") is eventually fleshed out into technical and/or transferrable skills. If so, code as appropriate and don't code the first statement as ambiguous skills.
Relevant to field of study (prf)	Element will provide useful skills, knowledge, or experiences or generally "be useful" to their current field of research, their future graduate research, or generally because the seminar, course, research, or exam is in their discipline of study. General notes of an element being relevant to

	"future research" is not coded, this is just for the specific subdiscipline of chemistry, not for research in general.
Not necessarily related to field (pnf)	Element will provide useful skills, knowledge, experiences or generally "be useful" even though it is not explicitly within their field of study. This commonly comes in the form of something like "even if it's not in my field..."
Relevant to career (prc)	Element will provide skills, knowledge, or experiences that will help them achieve the desired career. Cannot be that simply completing the element gets them closer to degree, which gives them eligibility for a career. "Future work" is generally not taken to mean career, but rather research. It must be a specific career or sector mentioned to qualify, otherwise it's "unrelated to career."
Not necessarily related to career (pnc)	Element will provide useful skills, knowledge, experiences or generally "be useful" even though they may not go into a specific career. This commonly comes in the form of something like "even if it's not the career I eventually take..." or "useful for any career."
Exciting, enjoyable, interesting (pee)	Element is described as exciting, enjoyable, interesting, and other positive adjectives that suggest the student experience positive affect in the element.
Important (pim)	Element is described as moderately, very, or the most important part of graduate education program, crucial, "the whole point", irremovable, has greater importance than other elements.
Good presentation/advisor (pgp)	When the instructor is described as a good teacher or passionate, helpful (courses); speaker is described as motivating, inspiring, good presenter (seminars); committee members or exam giver (not advisor, separate code) is described as caring, helpful; or advisor as good, helpful, caring, mentor, positive. For seminars, if someone says they learn from good and bad presenters, code as transferrable skills, pgp, and ngp.
Flexibility (pfl)	[Usually applies to courses and seminars] Offers flexibility in the courses, seminars, exams, and research experiences, which is viewed positively.
Positive (pos)	Any statement about an element that is positive, but cannot be classified into the other positive codes.

Networking (pne)	[Mostly applies to seminars] Element provides networking opportunities by meeting new people, interacting with other faculty and students. Does not have to explicitly tie to getting a career, could just be about making new connections to be a better researcher.
Upkeep (pup)	Element allows student to stay up to date on current research and know who is in the field and what they are doing. If the remark is specifically about gaining new methods and strategies, it should be coded as "Gain technical skills" and not here; this must be about who's who and what they are doing, not about new methods and things. However, if ambiguous like "keep up" and "stay up to date on the field", code here.

Negative Codes

Code	Description
No gain of skills (ambiguous, nsa)	Element did **not** provide a tangible quantity of skills, knowledge, or experiences. This is not broken into technical and transferrable because most students used broad statements like "I did not learn anything". "Tangible" quantity is indicated by adjectives like "a few", "no skills", or "a little". Because we asked students if it was a "waste of time" do not code this here, they are just telling us what they responded. Also, code here for "not useful" if we don't know if it's useless in terms of field or career, or if the student "does not remember" anything from classes.
Not relevant to field of study (nrf)	Element will **not** provide useful skills, knowledge, or experiences or generally "is not useful" to their current field of research, their future graduate research, or generally because the seminar, course, research, or exam is **not** in their discipline of study.
Not relevant to career (nrc)	Element will **not** provide skills, knowledge, or experiences that will help them achieve the desired career. Cannot be that simply completing the element gets them closer to degree, which gives them eligibility for a career.
Not exciting, enjoyable, interesting (nee)	Element is described as unenjoyable, boring, not interesting, and other negative adjectives that suggest the student experiences negative affect in the element.

Not important (nim)	Element is described as being of little importance or unnecessary part of graduate education program, wasteful, time is better spent elsewhere or the component took up too much time, has lesser importance than other elements, or is generally described as not useful.
Not good presentation/advisor (ngp)	When the instructor is described as a bad teacher, disinterested, uncaring, unhelpful, "there because they have to be" (courses); speaker is described as bad presenter, does not consider audience, too much jargon (seminars); committee members or exam giver (not advisor, separate code) is described as uncaring, just trying to fail student, or exams are not well-written; advisor in research uncaring, critical, bad, unhelpful, absent. For seminars, if someone says they learn from good and bad presenters, code as transferrable skills, pgp, and ngp.
No flexibility (nfl)	[Usually applies to courses and seminars and exams] Offers no or little flexibility in the courses, seminars, exams, and research experiences, which is viewed negatively. This is most commonly expressed when students are required to go to seminars or take courses and exams and speak about it negatively, or make it out to be a formality from their perspective.
Negative (neg)	Any statement about an element that is negative, but cannot be classified into the other negative codes.
Product (npr)	Element's benefit is constrained or evaluated only by some product of that element, like the number of papers published as a result of research, whether you passed/failed a test or course.
Bitter or extreme dissatisfaction (nbe)	Coded whenever a student says something that is more extreme than the average negative critique, indicated by using colorful or sarcastic phrasing, very clearly articulating hate or other strong emotions and dissatisfaction, or long-winded diatribes. Because we ask about wasting time, "waste of time" does not qualify for this code.
Mental health (nmh)	Coded whenever a student mentions a negative mental health from an element. Must be an explicit mention that this affected their mental health negatively, but the mental health can be as little as "harsh" or "stressful", does not have to be expressed with an adjective like "extreme". However, some view stress positively, which would be coded as soft/transferrable skills.

Not paying attention (npa)	[Most often applied to seminar and courses] Student reports to not pay attention in courses or seminars.
Not understanding (nun)	[Most often applied to seminar and courses] Student reports to not be able to understand the content of a seminar, course, or research field. Cannot simply say "it isn't in my subfield" (irrelevant field of study), but would need to explicitly mention or suggest that they don't understand it.

<u>Miscellaneous Codes</u>

Code	Description
Varying experiences (mve)	When the student mentions that benefits from the element are varying based on people involved and is phrased to not give any indication as to whether it is good or bad. This applies to speakers in seminars (e.g. "it depends on the speaker"), instructors in courses (e.g. "courses are hit or miss based on how the professor teaches it"), advisors in research and exams (e.g. "the research experience is highly dependent on the advisor"), or committee members and professors for exams (e.g. "some professors write good tests, others write bad ones"). To qualify, the student has to either leave out any negative/positive connotations (e.g. "it depends") or mention both negative and positive connotations (e.g. "some professors are good, others are bad"). If the latter is true, code as both good and not good presentation, if applicable. So "It depends, some are good teachers, some don't care" would be coded here; Good presentation and Not good presentation.
Unclear (mun)	No other codes can be applied, should be applied alone with no other codes.

Appendix B: Tests of Difference by Group

By primary division:

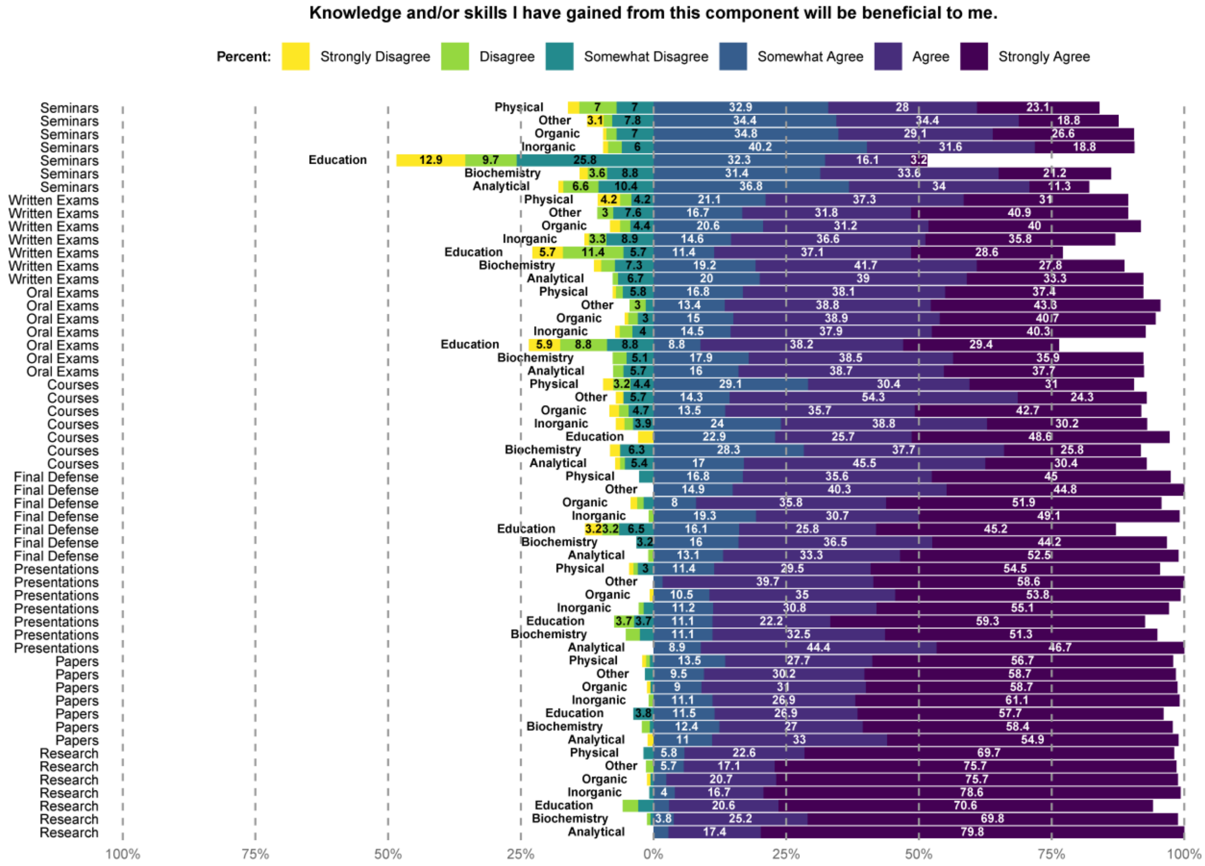

Appendix Figure 1: Overall benefits perceived by each element broken down by primary field of study.

By primary career interest:

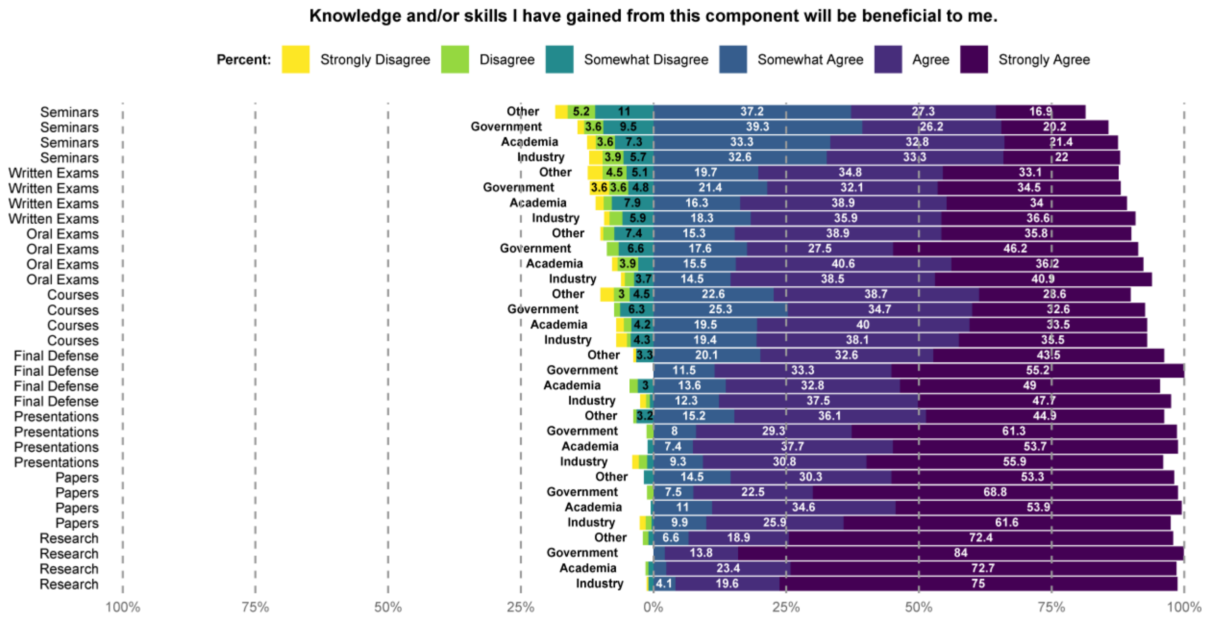

Appendix Figure 2:Overall benefits perceived by each element broken down by the sector a student's primary desired career resides

Statistical tests of difference (Kruskal-Wallis):

**Predictor:**	***Candidacy Status*[1]**			***Field***			***Desired Career***		
Question	χ^2	df	p*	χ^2	df	p*	χ^2	df	p*
Broadly beneficial, all elements									
15.a (courses broadly beneficial)	2.44	1	0.119	24.58	10	0.006*	10.59	4	0.032*
15.b (dissertation defense broadly beneficial)	7.46	1	0.006*	11.73	10	0.304	8.16	4	0.086
15.c (written exams broadly beneficial)	1.94	1	0.164	7.12	10	0.714	6.67	4	0.154
15.d (oral exams broadly beneficial)	1.60	1	0.205	10.01	10	0.439	4.51	4	0.341

15.e (seminars broadly beneficial)	9.68	1	0.002*	32.71	10	0.000*	14.67	4	0.005*
15.f (papers broadly beneficial)	0.30	1	0.587	7.40	10	0.687	7.44	4	0.114
15.g (presentations broadly beneficial)	0.03	1	0.859	11.30	9	0.256	9.30	4	0.054
15.h (research broadly beneficial	0.00	1	0.995	14.10	10	0.168	10.89	4	0.028*
Research, specific questions									
16.a (research is a waste of time)	0.51	1	0.475	16.66	10	0.082	7.67	4	0.104
16.b (gain transferrable skills from research)	0.94	1	0.333	23.23	10	0.010*	25.25	4	0.000*
16.c (research helps succeed in desired career)	0.83	1	0.362	15.17	10	0.126	28.48	4	0.000*
Courses, specific questions									
16.d (courses are a waste of time)	1.26	1	0.262	17.83	10	0.058	5.62	4	0.229
16.e (gain transferrable skills from courses)	3.49	1	0.062	21.70	10	0.017*	6.96	4	0.138
16.f (courses helps succeed in desired career)	1.99	1	0.158	16.92	10	0.076	6.12	4	0.190
Seminars, specific questions									
16.g (seminars are a waste of time)	2.33	1	0.127	23.88	10	0.008*	12.19	4	0.016*
16.h (gain transferrable skills from seminars)	5.77	1	0.016*	29.31	10	0.001*	5.90	4	0.207

16.i (seminars helps succeed in desired career)	6.38	1	0.012*	33.94	10	0.000*	5.74	4	0.219
Exams, specific questions									
16.j (exams are a waste of time)	0.22	1	0.636	13.94	10	0.176	8.56	4	0.073
16.k (gain transferrable skills from exams)	0.03	1	0.873	13.71	10	0.187	8.60	4	0.072
16.l (exams helps succeed in desired career)	0.19	1	0.667	14.61	10	0.147	11.95	4	0.018*

Appendix Table 1: Shows Kruskall-Wallis tests for differences between all characteristics predicted to systemically affect perceptions

[1]Candidacy Status defined by year: 2nd and 3rd year students were designated "pre candidacy" while 4th year + were "post." We are aware this is likely flawed as most students gain candidacy before the conclusion of their second year, but this is rather an indicator of whether the student has completed most of their programmatic requirements

Appendix C: Measurement Invariance

**Characteristic**	**Element**	**Measurement Invariance**	**Fit Stats**
Pre/Post Comp	Research	Base (Pre) ❌	did not converge
			Pre = 351, Post = 486
		Base (Post) ❌	X^2 (9.620), p (0.008), CFI (0.968), TLI (0.903), RMSEA (0.089), SRMR (0.131)
		Configural. ❌	did not converge
		Metric ✅	X^2 (12.082), p (0.098), CFI (0.994), TLI (0.990), RMSEA (0.042), SRMR (0.113)
		Scalar ✅	X^2 (11.135), p (0.084), CFI (0.994), TLI (0.988), RMSEA (0.046), SRMR (0.101), Δ CFI (0.000), ΔRMSEA (0.004), Δ SRMR (-0.012)
Pre/Post Comp	Course	Base (Pre) ✅	X^2 (2.079), p (0.354), CFI (1.000), TLI (1.000), RMSEA (0.011), SRMR (0.037)
		Base (Post) ✅	X^2 (3.568), p (0.168), CFI (0.999), TLI (0.997), RMSEA (0.040), SRMR (0.036)
		Configural ✅	X^2 (5.648), p (0.227), CFI (0.999), TLI (0.998), RMSEA (0.032), SRMR (0.036)
		Metric ✅	X^2 (7.362), p (0.392), CFI (1.000), TLI (1.000), RMSEA (0.011), SRMR (0.040), ΔCFI (0.001), ΔRMSEA (−0.021), ΔSRMR (0.004)
		Scalar ✅	X^2 (6.520), p (0.368), CFI (1.000), TLI (1.000), RMSEA (0.014), SRMR (0.036), ΔCFI (0.000), ΔRMSEA (0.003), ΔSRMR (0.004)
Pre/Post Comp	Exam	Base (Pre) ✅	X^2 (9.834), p (0.080), CFI (0.997), TLI (0.994), RMSEA (0.057), SRMR (0.092)
		Base (Post) ❌	X^2 (24.969), p (0.000), CFI (0.993), TLI (0.986), RMSEA (0.094), SRMR (0.087)
		Configural ❌	X^2 (34.803), p (0.000), CFI (0.995), TLI (0.989), RMSEA (0.081), SRMR (0.089)
		Metric ✅	X^2 (41.966), p 0.000), CFI (0.994), TLI (0.991), RMSEA (0.073), SRMR (0.095), ΔCFI (-0.001), ΔRMSEA (-0.008), ΔSRMR (0.006)
		Scalar ✅	X^2 (39.145), p (0.000), CFI (0.994), TLI (0.991), RMSEA (0.073), SRMR (0.092), ΔCFI (0.000), ΔRMSEA (0.000), ΔSRMR (-0.003)
Pre/Post Comp	Seminar	Base (Pre) ✅	X^2 (2.031), p (0.362), CFI (1.000), TLI (1.000), RMSEA (0.007), SRMR (0.030)

		Base (Post) ✅	X^2 (2.678), p (0.262), CFI (1.000), TLI (0.999), RMSEA (0.028), SRMR (0.027)
		Configural ✅	X^2 (4.709), p (0.318), CFI (1.000), TLI (0.999), RMSEA (0.022), SRMR (0.028)
		Metric ✅	X^2 (7.402), p (0.388), CFI (1.000), TLI (1.000), RMSEA (0.012), SRMR (0.035), ΔCFI (0.001), ΔRMSEA (-0.010), ΔSRMR (0.007)
		Scalar ✅	X^2 (4.878), p (0.560), CFI (1.000), TLI (1.001), RMSEA (0.000), SRMR (0.028), ΔCFI (0.000), ΔRMSEA (-0.012), ΔSRMR (-0.007)
Analy/Biochem/Ed/Inorg/Org/Phys/Other Field	Research	Base (Analy) ❌	ordered variable(s) has/have only 1 level
			Analy = 113, Biochem = 153, Ed=35, Inorg=127,Org=173, Phys=158, Other = 78
		Base (Biochem) ✅	X^2 (0.162), p (0.922), CFI (1.000), TLI (1.018), RMSEA (0.000), SRMR (0.023)
		Base (Ed) ❌	X^2 (7.380), p (0.025), CFI (1.000), TLI (0.999), RMSEA (0.286), SRMR (0.146)
		Base (Inorg) ✅	X^2 (0.004), p (0.998), CFI (1.000), TLI (1.004), RMSEA (0.000), SRMR (0.006)
		Base (Org) ❌	X^2 (14.013), p (0.001), CFI (0.955), TLI (0.864), RMSEA (0.189), SRMR (0.274)
		Base (Phys) ✅	X^2 (0.632), p (0.729), CFI (1.000), TLI (1.024), RMSEA (0.000), SRMR (0.056)
		Base (Other) ✅	X^2 (0.750), p (0.687), CFI (1.000), TLI (1.027), RMSEA (0.000), SRMR (0.058)
		Configural ❌	some variables have no variance in group 2
		Metric ❌	some variables have no variance in group 2
		Scalar ❌	some variables have no variance in group 2
	Course	Base (Analy) ✅	X^2 (0.494), p (0.781), CFI (1.000), TLI (1.010), RMSEA (0.000), SRMR (0.039)
		Base (Biochem) ✅	X^2 (0.845), p (0.655), CFI (1.000), TLI (1.007), RMSEA (0.000), SRMR (0.029)
		Base (Ed) ✅	X^2 (0.797), p (0.671), CFI (1.000), TLI (1.020), RMSEA (0.000), SRMR (0.044)
		Base (Inorg) ✅	X^2 (2.862), p (0.239), CFI (0.997), TLI (0.991), RMSEA (0.059), SRMR (0.062)

		Base (Org) ✅	X^2 (2.398), p (0.301), CFI (0.999), TLI (0.996), RMSEA (0.034), SRMR (0.051)
		Base (Phys) ✅	X^2 (1.245), p (0.537), CFI (1.000), TLI (1.007), RMSEA (0.000), SRMR (0.046)
		Base (Other) ✅	X^2 (0.309), p (0.857), CFI (1.000), TLI (1.007), RMSEA (0.000), SRMR (0.017)
		Configural ✅	X^2 (8.966), p (0.833), CFI (1.000), TLI (1.005), RMSEA (0.000), SRMR (0.043)
		Metric ✅	X^2 (7.362), p (0.392), CFI (1.000), TLI (1.000), RMSEA (0.011), SRMR (0.040), Δ CFI (0.000), ΔRMSEA (0.011), ΔSRMR (-0.003)
		Scalar ✅	X^2 (6.520), p (0.368), CFI (1.000), TLI (1.000), RMSEA (0.014), SRMR (0.036), ΔCFI (0.000), ΔRMSEA (0.003), ΔSRMR (-0.004)
	Exam	Base (Analy) ❌	did not converge
		Base (Biochem) ✅	X^2 (7.956), p (0.159), CFI (0.987), TLI (0.974), RMSEA (0.065), SRMR (0.118)
		Base (Ed) ✅	X^2 (2.074), p (0.839), CFI (1.000), TLI (1.025), RMSEA (0.000), SRMR (0.088)
		Base (Inorg) ✅	X^2 (4.221), p (0.518), CFI (1.000), TLI (1.000), RMSEA (0.000), SRMR (0.071)
		Base (Org) ❌	X^2 (13.620), p (0.018), CFI (0.996), TLI (0.992), RMSEA (0.106), SRMR (0.125)
		Base (Phys) ✅	X^2 (7.490), p (0.187), CFI (0.996), TLI (0.992), RMSEA (0.060), SRMR (0.102)
		Base (Other) ❌	X^2 (3.612), p (0.607), CFI (1.007), TLI (0.000), RMSEA (0.000), SRMR (0.088)
		Configural ❌	did not converge
		Metric ❌	X^2 (74.976), p (0.078), CFI (0.999), TLI (0.998), RMSEA (0.050), SRMR (0.135)
		Scalar ✅	X^2 (57.980), p (0.297), CFI (1.000), TLI (0.999), RMSEA (0.030), SRMR (0.115), Δ CFI (0.001), ΔRMSEA (-0.020), ΔSRMR (-0.020)
	Seminar	Base (Analy) ✅	X^2 (1.272), p (0.529), CFI (1.000), TLI (1.003), RMSEA (0.000), SRMR (0.033)
		Base (Biochem) ✅	X^2 (0.836), p (0.658), CFI (1.000), TLI (1.007), RMSEA (0.000), SRMR (0.026)
		Base (Ed) ✅	X^2 (0.643), p (0.725), CFI (1.000), TLI (1.017), RMSEA (0.000), SRMR (0.048)

		Base (Inorg) ✅	X^2 (1.199), p (0.549), CFI (1.000), TLI (1.011), RMSEA (0.000), SRMR (0.049)
		Base (Org) ✅	X^2 (1.078), p (0.583), CFI (1.000), TLI (1.008), RMSEA (0.000), SRMR (0.035)
		Base (Phys) ✅	X^2 (1.288), p (0.525), CFI (1.000), TLI (1.004), RMSEA (0.000), SRMR (0.034)
		Base (Other) ✅	X^2 (0.267), p (0.875), CFI (1.000), TLI (1.021), RMSEA (0.000), SRMR (0.024)
		Configural ❌	did not converge
		Metric ✅	X^2 (20.387), p (0.944), CFI (1.000), TLI (1.006), RMSEA (0.000), SRMR (0.058)
		Scalar ✅	X^2 (15.933), p (0.938), CFI (1.000), TLI (1.006), RMSEA (0.000), SRMR (0.037), ΔCFI (0.000), ΔRMSEA (0.000), ΔSRMR (-0.021
Acad/Govt/Ind /MDOther/Not SpecifiedPref Career	Research	Base (Acad) ✅	X^2 (1.117), p (0.572), CFI (1.000), TLI (1.025), RMSEA (0.000), SRMR (0.071)
			Acad=215, Govt = 95, Ind =301, MDOther = 200, NotSpecified = 26
		Base (Govt) ❌	ordered variable(s) has/have only 1 level
		Base (Ind) ✅	X^2 (1.577), p (0.45), CFI (1.000), TLI (1.005), RMSEA (0.000), SRMR (0.100)
		Base (MDOther) ✅	X^2 (0.207), p (0.902), CFI (1.000), TLI (1.006), RMSEA (0.000), SRMR (0.016)
		Base (NotSpecified) ❌	ordered variable(s) has/have only 1 level
		Configural ❌	some variables have no variance in group 2
		Metric ❌	some variables have no variance in group 2
		Scalar ❌	some variables have no variance in group 2
	Course	Base (Acad) ✅	X^2 (0.187), p (0.911), CFI (1.000), TLI (1.014), RMSEA (0.000), SRMR (0.017)
		Base (Govt) ✅	X^2 (1.836), p (0.399), CFI (1.000), TLI (1.003), RMSEA (0.000), SRMR (0.067)
		Base (Ind) ✅	X^2 (1.792), p (0.408), CFI (1.000), TLI (1.001), RMSEA (0.000), SRMR (0.029)

		Base (MDOther) ✅	X^2 (1.903), p (0.386), CFI (1.000), TLI (1.001), RMSEA (0.000), SRMR (0.040)
		Base (NotSpecified) ✅	X^2 (0.574), p (0.750), CFI (1.000), TLI (1.019), RMSEA (0.000), SRMR (0.046)
		Configural ✅	X^2 (6.291), p (0.790), CFI (1.000), TLI (1.005), RMSEA (0.000), SRMR (0.034)
		Metric ✅	X^2 (11.863), p (0.960), CFI (1.000), TLI (1.006), RMSEA (0.000), SRMR (0.051), ΔCFI (0.000), ΔRMSEA (0.000), ΔSRMR (0.017)
		Scalar ✅	X^2 (11.131), p (0.889), CFI (1.000), TLI (1.005), RMSEA (0.000), SRMR (0.039), ΔCFI (0.000), ΔRMSEA (0.000), ΔSRMR (-0.012)
	Exam	Base (Acad) ✅	X^2 (3.562), p (0.614), CFI (1.000), TLI (1.003), RMSEA (0.000), SRMR (0.053)
		Base (Govt) ✅	X^2 (2.706), p (0.745), CFI (1.000), TLI (1.007), RMSEA (0.000), SRMR (0.065)
		Base (Ind) ❌	X^2 (30.972), p (0.000), CFI (0.991), TLI (0.981), RMSEA (0.135), SRMR (0.144)
		Base (MDOther) ❌	X^2 (14.084), p (0.015), CFI (0.990), TLI (0.979), RMSEA (0.103), SRMR (0.117)
		Base (NotSpecified) ❌	X^2 (5.709), p (0.336), CFI (0.995), TLI (0.991), RMSEA (0.079), SRMR (0.177)
		Configural ❌	X^2 (57.033), p (0.000), CFI (0.994), TLI (0.988), RMSEA (0.092), SRMR (0.107)
		Metric ✅	X^2 (75.969), p (0.001), CFI (0.993), TLI (0.992), RMSEA (0.075), SRMR (0.115), ΔCFI (-0.001), ΔRMSEA (0-.017), ΔSRMR (0.008)
		Scalar ✅	X^2 (63.462), p (0.004), CFI (0.995), TLI (0.993), RMSEA (0.069), SRMR (0.107), ΔCFI (0.002), ΔRMSEA (0-.006), ΔSRMR (-0.008)
	Seminar	Base (Acad) ✅	X^2 (1.592), p (0.451), CFI (1.000), TLI (1.002), RMSEA (0.000), SRMR (0.033)
		Base (Govt) ✅	X^2 (0.276), p (0.871), CFI (1.000), TLI (1.022), RMSEA (0.000), SRMR (0.023)
		Base (Ind) ✅	X^2 (0.940), p (0.625), CFI (1.000), TLI (1.002), RMSEA (0.000), SRMR (0.019)
		Base (MDOther) ✅	X^2 (0.856), p (0.652), CFI (1.000), TLI (1.009), RMSEA (0.000), SRMR (0.026)
		Base (NotSpecified) ❌	Did not converge
		Configural ❌	did not converge

		Metric ✅	X^2 (19.475), p (0.616), CFI (1.000), TLI (1.001), RMSEA (0.000), SRMR (0.049)
		Scalar ❌	did not converge
